# The credibility crisis in research: Can economics tools help?

**DOI:** 10.1371/journal.pbio.2001846

**Published:** 2017-04-26

**Authors:** Thomas Gall, John P. A. Ioannidis, Zacharias Maniadis

**Affiliations:** 1 Economics Department, School of Social Sciences, University of Southampton, Southampton, United Kingdom; 2 Meta-Research Innovation Center at Stanford (METRICS), Stanford University, Stanford, California, United States of America

## Abstract

The issue of nonreplicable evidence has attracted considerable attention across biomedical and other sciences. This concern is accompanied by an increasing interest in reforming research incentives and practices. How to optimally perform these reforms is a scientific problem in itself, and economics has several scientific methods that can help evaluate research reforms. Here, we review these methods and show their potential. Prominent among them are mathematical modeling and laboratory experiments that constitute affordable ways to approximate the effects of policies with wide-ranging implications.

## Introduction

Serious worries have been voiced concerning a “reproducibility crisis” in many biomedical as well as social sciences; this crisis of confidence is fueled by the observation that numerous established findings may correspond to false positives that cannot be reproduced [1–5]. In response to the aforementioned concerns, several reforms have been put forward in various disciplines, purported to increase reproducibility [6]. Special focus has been placed on reforming researcher incentives [7,8,9], and some specific proposals have attracted considerable attention [10,11,12]. However, the study of behavioral responses to incentives is typically not the main focus of biomedical disciplines.

Behavioral responses to incentives may be evaluated with some modeling approaches followed in economics and related disciplines (e.g., political science). These disciplines have a policy focus, supported by the systematic study of how behavior responds to incentives. Formal economic tools are continually evolving and can be usefully employed for any policy analysis, but as yet they tend to be relatively unknown to the biomedical community. It is important to better understand these tools, especially when so many critical reforms of academic structures and incentives are being proposed. In this paper, our objectives are, first, to illustrate the possible benefits of economic analysis with concrete examples from existing reforms in which this analysis provides new insights and, second, to provide a relatively broad review of the relevant tools that can be employed to assess future reform proposals in biomedical sciences.

Although this review focuses on economics and related disciplines, some of the rigorous tools we review here are also used outside the social sciences (laboratory experimentation is common in psychology, game theory/dynamic modeling is widely used in evolutionary biology, and randomized controlled trials are common in clinical medicine). Clearly, relevant contributions from these disciplines will naturally be included in this review.

### Key concepts

Social phenomena exhibit a level of complexity and practical or ethical constraints that often make them not easily amenable to direct experimentation. However, the relevant problems may be approximated with mathematical modeling and empirical methods based on modeling. This approach has led to insightful conceptual developments that are worth summarizing.

#### Strategic interaction

The incentives that one individual faces depend on the expectations about others’ behavior, which in turn depends on their incentives and beliefs. A stylized model can illustrate the point: upon submitting an article for publication, a researcher has several possible “strategies.” In particular, she may opt to reveal all relevant details, gloss over important details, or even grossly falsify the evidence. Hence, implementing proposals that increase transparency (e.g., protocol preregistration, sharing of full data, etc.) will affect the relative benefit of each of these options. What each researcher is likely to do depends also on what she expects other researchers will do. “Game Theory” is the mathematical branch of economics that tackles interdependences of this sort.

#### Cost–benefit analysis

Economic models can explicitly address benefits and costs, including “opportunity costs.” Some reform proposals may become unattractive because of the accompanying costs. For example, considerable time and effort may be required to audit labs, replicate experiments, or meticulously prepare raw data for sharing. When this opportunity cost becomes too high, implementing transparency reforms might lead to a worse state of affairs. “Welfare Economics” systematically compares the costs and benefits for society resulting from a policy change.

#### Asymmetric information

Different actors in the scientific environment possess different kinds of useful information. This is important because some agents (funding agencies, the general public, etc.) wish to affect the behavior of others (researchers) with the purpose of achieving certain desirable outcomes—e.g., a greater overall rate of knowledge accumulation. An important branch of “Information Economics” is agency theory, which analyzes what a “principal” needs to consider in order to control the behavior of an “agent” who has superior information.

#### Public goods

When a certain action has a greater benefit for society as a whole than for the individual who chooses it, the action has characteristics of a “public good.” This is notable because in the presence of public goods, if everyone pursues their self-interest, society as a whole loses. In particular, scientific reproducibility can be viewed as a public good. Some scholars dispute that scientific knowledge is a public good, i.e., nobody can be excluded from its benefits. Instead, science may be a “contribution good,” since experts cannot be excluded from benefits but nonexperts can be [13].

#### Intellectual property versus free competition

The degree to which the government should grant legal protection of intellectual property may be decided based on economic arguments. Current research in biomedicine is often conducted by private entities (such as pharmaceutical companies or entrepreneurial start-ups). Given the obvious trade-off between transparency and trade secrecy, economic reasoning is required in order to analyze the arguments for “stealth research,” which is not shared with the wider scientific community [14].

### Mathematical modeling of incentives

Of course, tensions between individual and social objectives in the pursuit of science have been acknowledged and recognized for some time [15, 16]. Mathematical modeling can provide a rigorous framework for analyzing the potential effects of policy changes. Moreover, a good model may allow the analyst to uncover and specify mechanisms that would have been unclear otherwise. In particular, game theory is a useful tool to assess possible consequences of institutional reforms on individual incentives and aggregate outcomes.

To illustrate, consider a policy of strictly reporting research with perfect honesty, completeness, and thoroughness (e.g., fully implementing reporting guidelines such as CONSORT or Preferred Reporting Items for Systematic Reviews and Meta-Analyses [PRISMA] [17, 18], using proper statistical methods and reporting the full results). Such a policy would try to rule out “lying by omission” (e.g., not reporting all details of the design, especially those that may generate concerns about the study, or using questionable research practices [19,20] that will deliver seemingly more significant and seemingly more robust results) but not conscious overt fraud (e.g., fabrication of data, reporting nonexisting analyses). Assuming that such a policy will not be too cumbersome to implement and monitor (so that misleading omission will indeed be precluded), consider a model of competition for publishing mediated by scientific journals that was developed by Gall and Maniadis [21]. The model aims for simplicity rather than generality, but is well suited to demonstrate the working of game theoretic analysis, revealing the strategic interdependency between different activities that will determine what one should expect from different policies.

**Fig 1:**
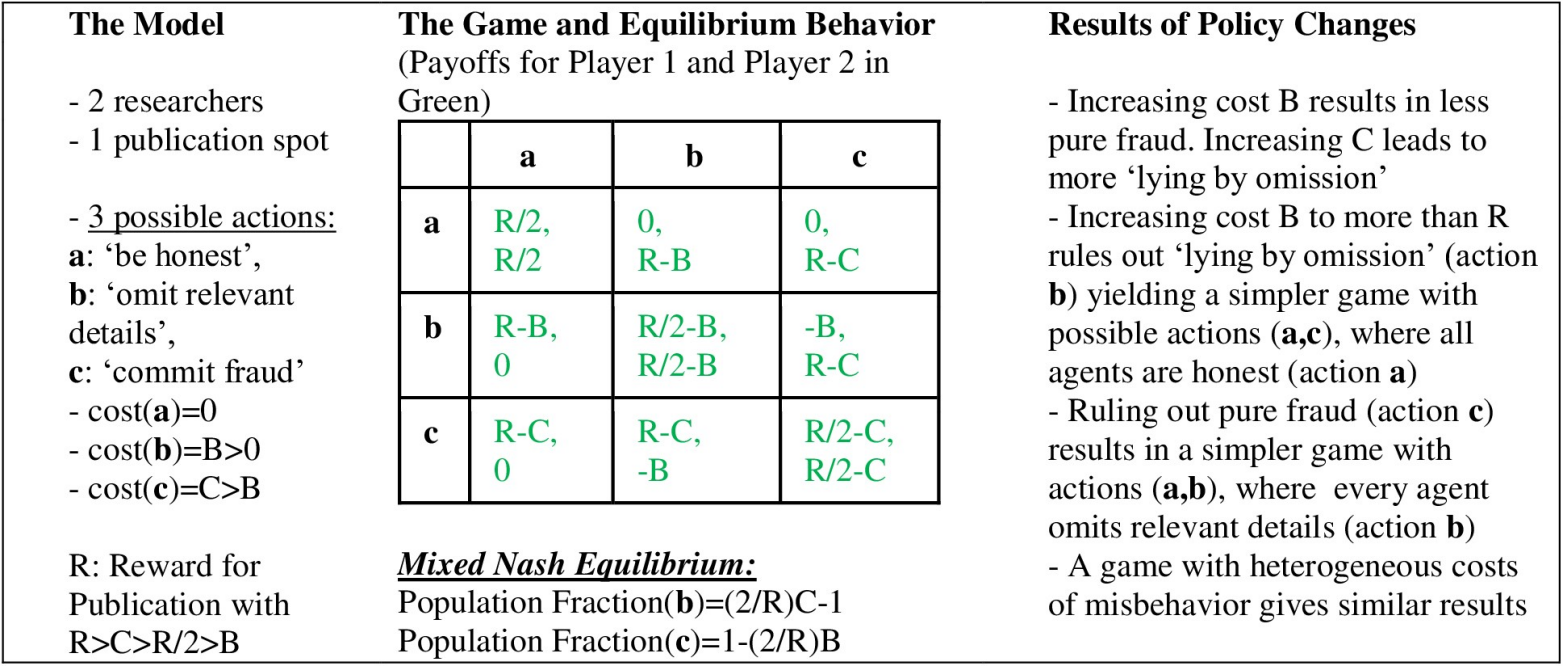
Modeling the consequences of reporting research with perfect honesty, omitting relevant details, or committing overt fraud.

As suggested by Stephan [22], academic competition can be modeled as a tournament. Assume that researchers compete for one publication spot, and they can spend effort on “sexing up” their result, engaging in either “lying by omission” or conscious fraud. A higher level of cheating offers an advantage in publishing but has higher cost. Nash equilibrium analysis tells us that preventing “mild cheating” will also decrease the frequency of “extreme” cheating and reduce questionable behavior in total. Such strategic complementarity is not uncommon and also appears in a number of other games, such as the well-known paper–scissors–rock game. The result is robust to changes in parameters and model specifications and would support the policy of full disclosure with maximal transparency (Fig 1). From a dynamic point of view, a lower prevalence of questionable behavior today yields more robust findings, which in turn will provide a more solid basis for future research. This will also affect the desirability of engaging in questionable behavior in the future, for instance, by increasing the potential for robust, significant results or raising the cost of questionable behavior.

Bobtcheff and colleagues [23] point to another detrimental effect of winner-takes-all contests in scientific research: intense competition for attention could lead researchers to compromise on quality in order to be the first to publish a new result. Indeed, recent contributions from rigorous population models using evolutionary tools indicate that small and poor designs tend to yield an advantage in the dynamic publication race [24,25]. The higher the reward for a successful publication, the higher the temptation is to engage in questionable activities. An editor or reader who is aware of this reasoning will therefore discount the evidence or have incentives to check the result more diligently. Lacetera and Zirulia [26] use a mathematical model of the interaction between a researcher and a recipient (e.g., editor or reader), allowing for monitoring by the latter. They find ambiguous effects of policies that reduce the cost of monitoring or increase the rewards of successful publication, depending on the precise parametrization of their model.

Discounting findings that are too good to be true lies at the heart of “persuasion games.” A persuasion game has two players: a “sender” that conveys verifiable information and the “receiver” of this information. In applications, the sender role could correspond to a researcher, a reviewer, or a journal, and the counterpart role of the receiver could correspond to a reviewer, an editor, or the general public/general readership, respectively. For instance, for clinical drug trials, their industry sponsors provide empirical evidence on the effectiveness of a drug to decision-makers, e.g., regulators who decide whether to license the drug or clinicians who ponder whether to use it with their patients. The sender has a private interest in convincing the receiver that a certain assertion (e.g., that a drug is effective) is true and may have some degrees of freedom in what information to convey. For instance, one may decide to take multiple looks at the data and stop clinical trials once a desired empirical result emerges or use more readily obtained favorable results from surrogate endpoints.

Milgrom [27] summarizes some basic insights from persuasion games. If the information that the sender could have sent is perfectly known, a rational receiver perfectly discounts the sender’s exaggeration and infers the actual information (this is called the “unravelling argument”). Thus, there is no need for external intervention to improve information sharing. Similarly, as for disclosing research procedures, the well-known unravelling results by Grossman and Milgrom [28, 29] would suggest that expert referees will infer the worst from a sender’s lack of transparency, which in turn disciplines the sender. Unfortunately, this is no longer true if the receiver is uncertain about what information the sender could have revealed and what remains opaque or hidden. This insight suggests that a useful policy for reducing false-positives might entail enhancing transparency about the researchers’ degrees of freedom.

The sender may also first determine how much research to perform and then what to disclose to the receiver, yielding incentives to conduct an excessive number of trials and to selectively report the best-looking results [30]. A rational receiver will realize this, and the sender will therefore anticipate that very powerful evidence will be needed to convince the receiver. In any equilibrium of the game, the sender will conduct too many trials reaching for the largest possible sample and will reveal all results. The ability to selectively report will induce excessive experimentation by the sender but will benefit society, as this extra knowledge is fully revealed. This result again relies crucially on the receiver’s rationality and his perfect knowledge of the sender’s preferences and his arsenal of questionable research practices. Otherwise, not all information is revealed in equilibrium. The sender may even opt to conceal some information that would otherwise serve his interests (in order to avoid revealing his preferences). In another interesting case, if the sender knows that with some probability he will face a naïve receiver (who takes the information at face value), mandatory disclosure is useful because the sender is likely to conceal some negative results. The effects of strategic interaction are subtle and often yield surprising policy implications, emphasizing the need for an explicit game-theoretic framework.

Ottaviani and colleagues [31,32] examine the optimal policies of receivers, such as regulatory authorities in drug approval procedures. Rational authorities will fully anticipate that any approval policy will induce the sender to respond strategically, e.g., by choosing the number of trials until a desirable empirical pattern emerges or fiddling with the assignment of subjects to treatment and control groups. In equilibrium, the authority has correct expectations on the sender’s manipulation and uses this information to interpret the results reported. If the players in this game are rational, the authority will correctly infer all information that is generated by the sender’s experimentation. Since the sender’s information is fully inferred by the receiver, the interesting question is whether certain rules, such as approval standards or transparency requirements, induce the sender to generate more or less information. For instance, Ottaviani and colleagues identify cases where commitment to well-defined approval standards can mitigate problems of excessive research.

Felgenhauer and Schulte [33] show that increasing the costs of presenting additional evidence can increase the informational value of a given set of evidence and can be socially beneficial because it “separates wheat from chaff.” Following this reasoning, the informational value of evidence may differ between different fields or journals, reflecting disparities in generating new evidence and the value of being published, respectively. This would suggest that in disciplines in which generating new evidence is cheap (or in disciplines in which articles tend to be submitted to a small number of elite journals, in which the possible reward is higher) standards should be more conservative and demanding than in fields in which generating evidence is more costly or the publication stakes are lower. This model thus suggests a surprising beneficial side-effect of raising the research documentation standards. The mathematical biology/ecology literature has also tackled the issue whether increasing the difficulty of publication (according to some criterion, i.e., statistical significance) could have beneficial effects. Some studies find that liming the communication of research findings can sometimes have beneficial effects on the informational value of observed results [34]. However, other studies find the opposite and argue that their conclusion is driven by the absence of an assumed explicit or implicit cost of publishing or reading articles [35].

Park, Peacey, and Munafò [36] point out that researchers learn about other informed agents’ opinions, adjusting their beliefs about the likely true answer to research questions. Such observational learning may lead to herding (relying more on other researchers’ opinions) and a loss of socially valuable information. Allowing reviewers to have a modicum of subjectivity in their recommendation may mitigate the problem. Accordingly, proposals for introducing a system to achieve more “mechanical decisions” at the review stage may have a negative effect by exacerbating herding.

There are many more issues in the design and analysis of research practices that mathematical modeling tools from economics and other disciplines could perhaps fruitfully address. Two examples are incentives in peer review and the role of intermediaries in science. Economic theory can improve our understanding of why incentives for referees are so low [37]. The literature on “platform competition” may be readily applied to examine the role that intermediaries (such as journals, editors, or publishing houses) may play in ensuring credibility of empirical research, for instance, in light of the emergence of open access journals [38].

### The role of the lab

In recent decades, controlled laboratory experiments have become more popular in economics. These experiments are typically computer-based, use a neutral framing (to avoid priming subjects), and offer nontrivial monetary incentives [39]. Plott [40] argues that the lab can be used as a “testbed” to address the effects of a policy change: “[…] first conduct experiments with a policy (preferably several competing policies) implemented in a simple environment. The outcomes are evaluated according to some pre-specified criteria, such as efficiency, which can be measured in an experimental environment. If performance is sufficiently bad, a policy is to be dropped, and if it shows promise, then the environment is complicated to offer the policy a more complex challenge.” The focus is not only on proof of principle but also on whether a given mechanism works for reasons consistent with the principles behind the mechanism’s design [41]. Roth [42] argues that “design economics” (a combination of economic theory, computation, and experiments) can be used to analyze and test the properties of new institutions.

The most well-known application in medicine might be Roth’s market-design approach for reforming the market for new physicians in the United States and Canada (Table 1). In the absence of centralized intervention, this market exhibited a natural inefficiency—the timing of agreements between new doctors and hospitals unraveled to increasingly early dates (even two years before the end of a physician’s training). Kagel and Roth [43] examined experimentally whether mechanisms with good theoretical properties are superior to those that lack such desirable properties. They found that lab behavior reproduces the evidence from natural settings, which lends support to the idea that it is the allocation mechanism that drives differences in the real world rather than uncontrolled differences across markets. Other examples of economic modeling successfully complemented by laboratory experiments include optimal auction design for radio spectrum licenses [44] and studying the consequences of issuing tradable “emission permits” to polluting companies [45].

**Table 1:** The market for new doctors: Economic modeling and experiments.

The Problem	Economic Method Used	Advantages of Methods	Weaknesses of Methods
• The previous system allocating new doctors to hospitals could be “gamed” by individual doctors and hospitals that find mutual gain in circumventing it. • A new scheme for matching supply with demand needed to be developed.	**Mathematical Modeling** **•** Mathematical analysis of the general properties of an allocation mechanism. **•** A given mechanism can be “unstable”: subgroups of individuals could reach mutually profitable arrangements outside the mechanism. **Economic Experiments** • Examination, in a simple environment, whether different allocative mechanisms are causally related to different outcomes.	**•** Theory and experiments indicate that the “deferred-acceptance algorithm” [46] yields a stable and efficient match of doctors and hospitals. No mutually beneficial outcome can be reached by circumventing the mechanism. Centralized clearinghouses have been organized around this concept. **•** The lab complements field evidence and helps resolve empirical debates about institutions.	**•** The real market was more complex than modeled (e.g., couples were often searching jointly). **•** External validity of experiments is a potential concern.

The combination of economic theory and laboratory experiments can be fruitfully applied to the problem of reforms in research. For example, policies that aim to alter practices at the journal or funder level are likely to have far-reaching “general equilibrium” effects. This means that entire markets will be affected by the policy change and often more than one market. For this reason, it is difficult for a randomized controlled trial to fully capture the relevant effects, and the economics lab can offer complementary evidence. Consider, for example, an editorial policy that makes mandatory the full documentation necessary for scientific reproducibility. When there is competition across journals, the response of other journals to the policy change will be critical. For example, suppose some journals adopt the policy (e.g., by requiring preregistration and full data and protocol sharing), but others do not. Then the proportion of papers allowing full reproducibility will increase in the former journals [47]. However, this does not imply that the proportion of such papers will increase across the entire field. Authors who benefit from these practices will send their papers preferentially to journals that have adopted the policy and avoid others. The whole “market” may not experience an increase in reproducible practices.

A randomized controlled trial at any given journal may then yield a misleading conclusion about the possible consequences of such policy changes. Economic modeling can help simulate the whole “market,” and lab experiments, complemented by rigorous field evidence, can provide useful insights. Theoretical analysis can also identify the likely intensity of a policy intervention required, depending on observable circumstances. For instance, when competition among journals undermines propping up reproducibility, a coordinated, centralized solution is needed. This can be achieved, for example, if authorities such as promotion committees and scientific associations recognize and offer more credit for publications in journals that impose high reproducibility standards. This will induce all journals to shift to a new regime in a concerted manner.

Moreover, most research funders are interested in the consequences of their policies according to some criterion, for instance, aiming to maximize the volume of reproducible knowledge from the activities that they support. It might be too costly for them to initiate their assessment by performing a randomized trial. However, they may use economic modeling and the laboratory to attack the problem in a simplified form before embarking on a decision to conduct a costly randomized trial or to scale up a policy plan.

### Testing models of researcher incentives

Laboratory experiments in economics can also inform realistic mathematical models. Almost all of the persuasion models described above assume that agents would happily deceive others if that would suit their own interests. However, introspection and morality suggest that this might not necessarily be the case. Indeed, while early economics experiments found that more than half of subjects lie often [48,49], many subjects do not lie fully, and the extent of alignment of incentives between the deceiver and the deceived also matters. Hence, there is a need to estimate the precise psychic costs of deceiving.

Fischbacher and Föllmi‐Heusi [50] use an experimental design that allows for more honest revelation of pure aversion to lying, net of social influences. About half of participants (students from Zurich) lie in the experiment, with about 22% doing so “completely.” In contrast, when using a similar experimental design for a representative sample of the German population, almost no participants chose to lie [51]. In a recent meta-analysis of experiments sharing this design, Abeler and colleagues [52] found that subjects forego about three-fourths of the potential gains from lying. Gneezy and colleagues [53] categorize behavior into different types and find that lying is increasing in its benefit and shows a small tendency to increase over time. A third of subjects in each period opt to always reveal the truth, while 28% choose the money-maximizing strategy.

Psychological experimental studies of unethical behavior focus less on measurement of aggregate cheating and more on revealing the complex nature of behavior under ethical dilemmas. This literature has taught us important lessons. Research misbehavior is likely to take place in a “group setting” (that allows diffusion of responsibility), and it concerns particularly creative people. Both factors tend to be associated with higher tendencies to engage in immoral behavior [54,55]. Moreover, observation of others’ cheating behavior tends to increase our own but only when the perpetrator is identified as an “in-group” member [56]. This points to the need of additional research on how scientists identify with certain groups.

This type of experimental evidence is complementary to surveys that tackle scientific misbehavior directly but face possible misrepresentation biases. Fanelli (see [20]) summarizes findings from several disciplines: a majority of researchers are involved in some type of questionable practices, although only 3% admit falsifying or fabricating data. There is a clear need for more survey and experimental evidence that employs researchers as participants and concentrates on a scientific context. An example of such an approach is the recent psychological research by Bakker and colleagues [57], who show that research psychologists have a flawed intuition about the power of their research designs.

In summary, laboratory experiments using economic tools hold a double promise. First, they can be used as simple tests of the viability and efficiency of alternative scientific practices (often complementing field evidence). Second, they may illuminate principles of human behavior that are likely to underlie behavior in the research environment and thus inform formal theories of such behavior.

### Empirical approaches in the field

A greater challenge is the identification of the quantitative causal effect of a policy on outcomes of interest in situ, that is, in the field rather than in the lab. As in the lab, the empirical setup in economics will usually rely on predictions from mathematical models. Two broad approaches are widely used: first, in a quasi or natural experiment, one might use naturally occurring variation in exposure to a policy of interest, if the variation in exposure is statistically independent of the outcomes of interest. Second, the researcher can conduct a randomized controlled trial. Randomized controlled trials have gained considerable popularity also in economics and, in particular, among those who examine the effects of social and economic policy interventions on a variety of individual and aggregate outcomes [58].

Economics lacks a long tradition of empirical studies that test the efficacy of peer review—with the exception of an early randomized controlled trial on double-blind refereeing [59]. In medicine, the problem has attracted attention since the late 1980s [60]. A recent synthesis of randomized controlled trials on the efficiency of peer review in biomedical research traced 21 articles [61]. The review categorized five types of interventions: (1) training or mentoring reviewers, (2) adding special peer reviewers such as statisticians, (3) peer reviewers’ use of a checklist, (4) open peer review, and (5) blinded peer review. The meta-analysis found weak average treatment effects for most interventions and concluded that evidence-based peer review needs to be developed further in biomedical journals. From this literature, we have learned much about the efficiency of the current system. We shall now argue that in some cases economic insights can advance the information that can be deduced from field evidence by enhancing our research designs.

The key idea is that economic models deliver quantitative hypotheses that can be tested and, more importantly, offer guidance as to which potential effects may in fact be generated by an intervention and should be examined. Since socio-economic systems of interactions are complex, an intervention likely triggers indirect effects as well as direct ones. Such indirect effects will not necessarily be the ones expected by naïve, informal reasoning alone (e.g., there might be “general equilibrium” effects as other agents react to an initial behavioral change triggered by an intervention). Nor will indirect effects necessarily be quantitatively less important than direct effects; this is precisely the type of question one may hope to answer empirically.

Let us illustrate the point using a particular example: open peer review (revealing the names of reviewers). Walsh and colleagues [62] show that open peer review has a small positive effect (which does not reach the level preassigned as “editorially significant”) on the quality of the reports. Furthermore, signed reviews are more courteous and take more time to prepare. Similarly, weak effects of open peer review have been found in other studies in biomedicine [63,64]. A particular difficulty arises when, on the basis of randomized controlled trials, general lessons for alternative systems need to be drawn. For example, it may be necessary to implicitly hypothesize that, in a system of open peer review, the loss of volunteering referees will be similar as the one measured in a given study. But how will reviewers’ volunteering behavior change if many/most journals use open peer review? How will the dynamics of article submissions change if some competitive journals use open peer review and some do not? Social science tools can help with this type of analysis, examining behavioral underpinnings of possible responses and the market-level interaction among journals. Such analysis can be incorporated in the article that presents the study and inform its design, for instance, by pointing to the need to measure other outcomes. These may include possible changes in the quantity and quality of submissions across journals using different policies but also in the quantity and quality of reviews for journals other than the ones assigned to the “treatment” (open review) and “control” groups of the trial.

For a concrete example, consider an economics study examining the impact of different editorial policies on review time [65]. The study considered two alternative predictions based on economic and behavioral insights. On the one hand, since economic incentives matter, setting deadlines and rewarding referees financially should improve the turnaround times. On the other hand, behavioral economics allows for the possibility that paying referees will “crowd out” intrinsic motivation [66]. That is, offering monetary payments emphasizes the pure profit motive for doing a task, possibly at the cost of reducing altruistic or civic duty motives. Based on this theoretical reasoning, the authors chose not only to include treatments that were designed to measure whether a policy “worked” but also tried to disentangle the differential theoretical pathways, to enhance generalizability. In particular, both economic and moral incentives were considered as treatments. Moreover, on top of standard outcome measures (such as review duration and length of reports), the authors measured average review durations at other economics journals at the same publisher to capture market-level effects. The results suggest that nudging and monetary incentives work well for economists, while publicizing an individual reviewer’s performance online only appears to affect more senior (tenured) reviewers moderately.

To further illustrate the methodology for accounting for market-level effects, consider the work of Card and Dellavigna [67], who examine the introduction of page limits at two established economics journals. What is of key importance is to get a quantitative flavor of the tendency of authors to substitute among journals. They compared the pattern of submitted article lengths before and after the policy change and were able to estimate the degree to which authors turned to different journals. To achieve this, they analyzed the incentives of authors and used the concept of “match surplus”: “the gap in payoffs between submission to the journal in question and the payoff to the next best alternative outlet.” Their analysis showed that the page policy did not change the supply of submitted articles for a top journal (American Economic Review), but it did reduce submissions when applied at a journal outside the elite “top-5” journals (the Journal of the European Economic Association). This suggests that the policy is only effective for a top journal, as authors will prefer to shorten their manuscripts, but, otherwise, they will submit elsewhere.

## Conclusions

There is a wealth of experience in economics and related social sciences when it comes to evaluating policy and changes in various practices. Economic theory is potentially useful as a pointer for empirical work but also in designing rules of the game under which science plays out. However, there may be a sizable gap between the economic wisdom on how best to organize the production of new knowledge and the discourse in other disciplines regarding how to address the credibility problem. Bridging this gap promises to generate what economists call a “Pareto improvement,” a better outcome for all parties involved.

Table 2 summarizes the methodologies we have discussed, along with their expected costs and benefits. In terms of the limitations of mathematical modeling, the role of simplifying assumptions is a major one. In particular, results may critically depend on the underlying assumptions of each model, and they must be laid down in a clear way. If models with alternative assumptions tend to point in the same direction, confidence in these results increases. In addition, some of the assumptions are hard to test or to verify, especially regarding preferences and individuals’ rationality.

**Table 2:** A taxonomy of economic methods.

Approach	Benefits	Concerns
**Mathematical Modeling**	**•** Can help analyze complex phenomena. **•** Guides empirical designs.	**•** Results based on specific assumptions. **•** Often difficult to directly test empirically.
**Economic Laboratory**	**•** Can be used to validate mathematical theories. **•** Relatively low-cost interventions.	**•** Based on limited samples and specific subject pools. **•** Hawthorne effects (being subject to an experiment alters behavior).
**Economic Empirical Approaches**	**•** Take into account market-level phenomena. **•** Can enhance the design of randomized controlled trials.	**•** So far, limited application on the domain of science.

The experimental approach also has important limitations and trade-offs. Often the results are based on convenience samples drawn from student populations and carried out in artificial environments (computer labs). For these reasons, the implications are more credible when fundamental aspects of behavior are tested, which are less dependent on context, experience, focused expertise, and demographics. Importantly, reservations about the external validity of experiments can be addressed by further experiments using more representative participant pools or approximating the real environment in some key dimensions. However, using more representative samples and more natural environments increases the cost, both in terms of money and experimental control. The optimal choice depends on the nature of the problem (degree of likely context dependence, etc.) and the cost of using natural populations/environments. Finally, although the empirical techniques for field evidence can be useful, their application to randomized controlled trials in science has been rudimentary, and they are still unproven.

While each of the methods has its own strengths and weaknesses, the fact that these strengths and weaknesses are heterogeneous and nonoverlapping reaffirms the potential benefits of complementarity. In practice, triangulation of a given result using the different methodologies could give us greater confidence in the assessment of a proposed intervention.

## References

[1] Ioannidis JP. Why science is not necessarily self-correcting. Perspect Psychol Sci. 2012 11 1;7(6):645–54. doi: 10.1177/1745691612464056 26168125 10.1177/1745691612464056

[2] Open Science Collaboration. Estimating the reproducibility of psychological science. Science. 2015 8 28;349(6251):aac4716 doi: 10.1126/science.aac4716 26315443 10.1126/science.aac4716

[3] Bettis RA. The search for asterisks: Compromised statistical tests and flawed theories. Strategic Management Journal. 2012 1 1;33(1):108–13.

[4] Brodeur A, Lé M, Sangnier M, Zylberberg Y. Star wars: The empirics strike back. Am Econ J Appl Econ. 2016 1 1;8(1):1–32.

[5] Chang AC, Li P. Is economics research replicable? Sixty published papers from thirteen journals say ‘usually not’ Washington: Board of Governors of the Federal Reserve System Finance and Economics Discussion Series 2015–083. https://papers.ssrn.com/sol3/papers.cfm?abstract_id=2669564. Cited 16 March 2017.

[6] Goodman SN, Fanelli D, Ioannidis JP. What does research reproducibility mean? Sci Transl Med. 2016 6 1;8(341):341ps12 doi: 10.1126/scitranslmed.aaf5027 27252173 10.1126/scitranslmed.aaf5027

[7] Ioannidis JP. How to make more published research true. PLoS Med. 2014 10 21;11(10):e1001747 doi: 10.1371/journal.pmed.1001747 25334033 10.1371/journal.pmed.1001747 PMC4204808

[8] Bakker M, van Dijk A, Wicherts JM. The rules of the game called psychological science. Perspect Psychol Sci. 2012 11;7(6):543–54. doi: 10.1177/1745691612459060 26168111 10.1177/1745691612459060

[9] Nosek BA, Spies JR, Motyl M. Scientific utopia: II. Restructuring incentives and practices to promote truth over publishability. Perspect Psychol Sci. 2012 11; 7(6):615–31. doi: 10.1177/1745691612459058 26168121 10.1177/1745691612459058 PMC10540222

[10] Fanelli D. Redefine misconduct as distorted reporting. Nature. 2013 2 14; 494(7436):149 doi: 10.1038/494149a 23407504 10.1038/494149a

[11] Simmons JP, Nelson LD, Simonsohn U. False-positive psychology: Undisclosed flexibility in data collection and analysis allows presenting anything as significant. Psychol Sci. 2011 11; 22(11):1359–66. doi: 10.1177/0956797611417632 22006061 10.1177/0956797611417632

[12] Landis SC, Amara SG, Asadullah K, Austin CP, Blumenstein R, Bradley EW, et al A call for transparent reporting to optimize the predictive value of preclinical research. Nature. 2012 10 11; 490(7419):187–91. doi: 10.1038/nature11556 23060188 10.1038/nature11556 PMC3511845

[13] Kealey T, Ricketts M. Modeling science as a contribution good. Res Policy. 2014 7 31; 43(6):1014–24.

[14] Ioannidis JP. Stealth research: is biomedical innovation happening outside the peer-reviewed literature? JAMA. 2015 2 17; 313(7):663–4. doi: 10.1001/jama.2014.17662 25688775 10.1001/jama.2014.17662

[15] Dasgupta P, David PA. Toward a new economics of science. Res Policy. 1994 9 30; 23(5):487–521.

[16] Kitcher P. The division of cognitive labor. J Philos. 1990 1 1;87(1):5–22.

[17] Schulz KF, Altman DG, Moher D. CONSORT 2010 statement: updated guidelines for reporting parallel group randomised trials. BMC Med. 2010 3 24; 8(1):18. 20334633 10.1186/1741-7015-8-18 PMC2860339

[18] Moher D, Liberati A, Tetzlaff J, Altman DG, Prisma Group. Preferred reporting items for systematic reviews and meta-analyses: the PRISMA statement. PLoS Med. 2009 7 21; 6(7):e1000097 doi: 10.1371/journal.pmed.1000097 19621072 10.1371/journal.pmed.1000097 PMC2707599

[19] John LK, Loewenstein G, Prelec D. Measuring the prevalence of questionable research practices with incentives for truth telling. Psychol Sci. 2012 4 16:0956797611430953. 10.1177/0956797611430953 22508865

[20] Fanelli D. How many scientists fabricate and falsify research? A systematic review and meta-analysis of survey data. PLoS ONE. 2009 5 29;4(5):e5738 doi: 10.1371/journal.pone.0005738 19478950 10.1371/journal.pone.0005738 PMC2685008

[21] Gall T, Maniadis Z. Evaluating solutions to the problem of false positives. University of Southampton Discussion Paper in Economics and Econometrics. 2015;1504. http://www.southampton.ac.uk/economics/research/discussion_papers/author/thomas_gall/1504-evaluating-solutions-to-the-problem-of-false-positives.page. Cited 16 March 2017.

[22] Stephan PE. How economics shapes science Cambridge, MA: Harvard University Press; 2012 1 15.

[23] Bobtcheff C, Bolte J, Mariotti T. Researcher’s dilemma. Rev Econ Stud. 2016 7 10:rdw038.

[24] Higginson AD, Munafò MR. Current incentives for scientists lead to underpowered studies with erroneous conclusions. PLoS Biol. 2016 11 10; 14(11):e2000995 doi: 10.1371/journal.pbio.2000995 27832072 10.1371/journal.pbio.2000995 PMC5104444

[25] Smaldino PE, McElreath R. The natural selection of bad science. R Soc Open Sci. 2016 9 1;3(9):160384 doi: 10.1098/rsos.160384 27703703 10.1098/rsos.160384 PMC5043322

[26] Lacetera N, Zirulia L. The economics of scientific misconduct. J Law Econ Organ. 2011 10 1; 27(3):568–603.

[27] Milgrom P. What the seller won't tell you: Persuasion and disclosure in markets. J Econ Perspect. 2008; 22(2): 115–131.

[28] Grossman SJ. The informational role of warranties and private disclosure about product quality. J Law Econ. 1981 12 1; 24(3):461–83.

[29] Milgrom PR. Good news and bad news: Representation theorems and applications. The Bell Journal of Economics. 1981 10 1:380–91.

[30] Henry E. Strategic disclosure of research results: The cost of proving your honesty. Econ J. 2009 7 1; 119(539):1036–64.

[31] Henry E, Ottaviani M. Research and the approval process. 2014; Paper presented at the Fifteenth CEPR/JIE Conference on Applied Industrial Organization, Athens, Greece, 21 to 24 May 2014. https://pdfs.semanticscholar.org/07f5/35ee8313a0b2fa3ec801f45336dfd2cd32fd.pdf. Cited 16 March 2017.

[32] Di Tillio A, Ottaviani M, Sorensen PN. Persuasion Bias in Science: Can Economics Help? CEPR Discussion Paper Series. 2016; DP11343. https://papers.ssrn.com/sol3/papers.cfm?abstract_id=2801004. Cited 16 March 2017.

[33] Felgenhauer M, Schulte E. Strategic private experimentation. Am Econ J Microecon. 2014; 6(4): 74–105.

[34] McElreath R, Smaldino PE. Replication, communication, and the population dynamics of scientific discovery. PLoS ONE. 2015 8 26; 10(8):e0136088 doi: 10.1371/journal.pone.0136088 26308448 10.1371/journal.pone.0136088 PMC4550284

[35] Nissen SB, Magidson T, Gross K, Bergstrom CT. Publication bias and the canonization of false facts. Elife. 2016 12 20; 5:e21451 doi: 10.7554/eLife.21451 27995896 10.7554/eLife.21451 PMC5173326

[36] Park IU, Peacey MW, Munafò MR. Modeling the effects of subjective and objective decision making in scientific peer review. Nature. 2014 2 6;506(7486):93–6. doi: 10.1038/nature12786 24305052 10.1038/nature12786

[37] Engers M, Gans JS. Why referees are not paid (enough). Am Econ Rev. 1998; 88(5): 1341–1349.

[38] McCabe MJ, Snyder CM, Fagin A. Open access versus traditional journal pricing: Using a simple “platform market” model to understand which will win (and which should). The Journal of Academic Librarianship. 2013; 39(1): 11–19.

[39] Hertwig R, Ortmann A. Experimental practices in economics: A methodological challenge for psychologists? Behav Brain Sci. 2001 6 1;24(03):383–403. 11682798 10.1037/e683322011-032

[40] Plott CR. Market architectures, institutional landscapes and testbed experiments. Econ Theory. 1994; 4.1, 3–10.

[41] Gillen BJ, Plott CR, Shum M. A Parimutuel-like Mechanism for Information Aggregation: A Field Test inside Intel. California Institute of Technology Social Science Working Paper Series. 2014; No. 1367. https://papers.ssrn.com/sol3/papers.cfm?abstract_id=2504171. Cited 16 March 2017.,

[42] Roth AE. The economist as engineer: Game theory, experimentation, and computation as tools for design economics. Econometrica. 2002 7 1; 70(4):1341–78.

[43] Kagel JH, Roth AE. The dynamics of reorganization in matching markets: A laboratory experiment motivated by a natural experiment. Q J Econ. 2000 2 1; 115(1):201–35.

[44] Ledyard JO, Porter D, Rangel A. Experiments testing multiobject allocation mechanisms. J Econ Manag Strategy. 1997 9 1; 6(3):639–75.

[45] Cason TN, Gangadharan L, Duke C. Market power in tradable emission markets: a laboratory testbed for emission trading in Port Phillip Bay, Victoria. Ecol Econ. 2003 10 31; 46(3):469–91.

[46] Gale D, Shapley LS. College admissions and the stability of marriage. Am Math Month. 1962 1 1; 69(1):9–15.

[47] Kidwell MC, Lazarević LB, Baranski E, Hardwicke TE, Piechowski S, Falkenberg LS, et al Badges to acknowledge open practices: A simple, low-cost, effective method for increasing transparency. PLoS Biol. 2016 5 12;14(5):e1002456 doi: 10.1371/journal.pbio.1002456 27171007 10.1371/journal.pbio.1002456 PMC4865119

[48] Gneezy U. Deception: The role of consequences. Am Econ Rev. 2005 3 1; 95(1):384–94.

[49] Sutter M. Deception through telling the truth?! Experimental evidence from individuals and teams. Econ J. 2009 1 1; 119(534):47–60.

[50] Fischbacher U, Föllmi‐Heusi F. Lies in disguise—an experimental study on cheating. J Eur Econ Assoc. 2013 6 1; 11(3):525–47.

[51] Abeler J, Becker A, Falk A. Representative evidence on lying costs. J Public Econ. 2014 5 31; 113:96–104.

[52] Abeler J, Nosenzo D, Raymond C. Preferences for truth-telling. CESifo Working Paper Series. 6087. https://papers.ssrn.com/sol3/papers.cfm?abstract_id=2866381. Cited on 16 March 2017.

[53] Gneezy U, Rockenbach B, Serra-Garcia M. Measuring lying aversion. J Econ Behav Organ. 2013 9 30; 93:293–300.

[54] Ayal S, Gino F. Honest rationales for dishonest behavior. The social psychology of morality: Exploring the causes of good and evil Washington, DC: American Psychological Association 2011 pp. 149–66.

[55] Gino F, Ariely D. The dark side of creativity: original thinkers can be more dishonest. J Pers Soc Psychol. 2012 3; 102(3):445 doi: 10.1037/a0026406 22121888 10.1037/a0026406

[56] Gino F, Ayal S, Ariely D. Contagion and differentiation in unethical behavior the effect of one bad apple on the barrel. Psychol Sci. 2009 3 1; 20(3):393–8. doi: 10.1111/j.1467-9280.2009.02306.x 19254236 10.1111/j.1467-9280.2009.02306.x

[57] Bakker M, Hartgerink CH, Wicherts JM, van der Maas HL. Researchers’ intuitions about power in psychological research. Psychol Sci. 2016 8 1; 27(8):1069–77. doi: 10.1177/0956797616647519 27354203 10.1177/0956797616647519 PMC4976648

[58] Duflo E, Glennerster R, Kremer M. Using randomization in development economics research: A toolkit In: Schultz TP, Strauss J, editors. Handbook of development economics Volume 4. Amsterdam: North-Holland, 2008 pp. 3895–962.

[59] Blank RM. The effects of double-blind versus single-blind reviewing: Experimental evidence from the American Economic Review. Am Econ Rev. 1991 12 1:1041–67.

[60] Rennie D. Guarding the guardians: a conference on editorial peer review. JAMA. 1986 11 7; 256(17):2391–2. 3773144

[61] Bruce R, Chauvin A, Trinquart L, Ravaud P, Boutron I. Impact of interventions to improve the quality of peer review of biomedical journals: a systematic review and meta-analysis. BMC Med. 2016 6 10; 14(1):85 doi: 10.1186/s12916-016-0631-5 27287500 10.1186/s12916-016-0631-5 PMC4902984

[62] Walsh E, Rooney M, Appleby L, Wilkinson G. Open peer review: a randomised controlled trial. Br J Psychiatry. 2000 1 1; 176(1):47–51. 10789326 10.1192/bjp.176.1.47

[63] van Rooyen S, Delamothe T, Evans SJ. Effect on peer review of telling reviewers that their signed reviews might be posted on the web: randomised controlled trial. BMJ. 2010 11 16; 341:c5729 doi: 10.1136/bmj.c5729 21081600 10.1136/bmj.c5729 PMC2982798

[64] Van Rooyen S, Godlee F, Evans S, Black N, Smith R. Effect of open peer review on quality of reviews and on reviewers' recommendations: a randomised trial. BMJ. 1999 1 2; 318(7175):23–7. 9872878 10.1136/bmj.318.7175.23 PMC27670

[65] Chetty R, Saez E, Sándor L. What policies increase prosocial behavior? An experiment with referees at the Journal of Public Economics. J Econ Perspect. 2014 7 1;28(3):169–88.

[66] Frey BS, Oberholzer-Gee F. The cost of price incentives: An empirical analysis of motivation crowding-out. Am Econ Rev. 1997 9 1; 87(4):746–55.

[67] Card D, DellaVigna S. Page limits on economics articles: Evidence from two journals. J Econ Perspect. 2014 7 1; 28(3):149–67.

